# Data from a multidisciplinary poll of 178 expert physicians on the usage of non-vitamin K Oral Anticoagulants in patients with atrial fibrillation and venous thromboembolism

**DOI:** 10.1016/j.dib.2017.09.064

**Published:** 2017-10-06

**Authors:** Paolo Colonna, Felicita Andreotti, Walter Ageno, Vittorio Pengo, Niccolò Marchionni

**Affiliations:** a Cardiology Department, Policlinico of Bari Hospital, Bari, Italy; b Dept of Cardiovascular Medicine, Catholic University, Rome, Italy; c Department of Clinical and Experimental Medicine, University of Insubria and Ospedale di Circolo, Varese, Italy; d Department of Cardiac, Thoracic, and Vascular Sciences, Padua University Hospital, Padua, Italy; e Department of Clinical and Experimental Medicine, Syncope Unit, and Division of General Cardiology, University of Florence and AOU Careggi, Florence, Italy

## Abstract

This data article contains data from a multidisciplinary questionnaires filled in by 178 expert physicians on the usage of non-vitamin K Oral Anticoagulants in patients with atrial fibrillation (AF) and for the treatment of patients with venous thromboembolism (VTE). The questionnaire consists of 9 statements of clinical complex AF and VTE cases and informative campaign on antithrombotic therapy for stroke prevention in AF. The data are potentially valuable for the scientific community, showing the doubts of different specialists (Internists, Pneumologists, Geriatricians, Cardiologists and Neurologists) with a large experience in prescribing oral anticoagulation in difficult AF and VTE cases (see full list of participants provided). The data obtained in some particular clinical cases such as CHA2DS2-VASc=1, comorbid coronary artery disease, frailty, advanced age, risk of falling and prior haemorrhagic stroke, can be compared with indications from published guidelines and recommendations for future insight and to be considered as a benchmark for future trials in the area or oral anticoagulation for AF and VTE.

The data concerning informative campaign on antithrombotic therapy for stroke prevention showed the expert panel agreement on the inclusion of self monitoring of heart rhythm by pulse taking in subjects older than 64 years of age (81% agreement, item 3); knowledge that the risk of stroke associated with AF is almost twice the risk associated with hypertension (95% agreement, item 4); knowledge that the CHA2DS2-VASc score exerts a higher influence on stroke risk compared to AF duration (92% agreement, item 5); knowledge that stroke prevention in AF with a NOAC is more effective, does not cause any higher bleeding risk, and is equally simple compared to aspirin treatment (91% agreement, item 6).

Data on strategies to optimise appropriate prescription of antithrombotic therapy showed agreement on the utility of short television advertisements about the risks of stroke associated with AF (79% agreement, item 8), on a campaign encouraging regular control of cardiac rhythm by pulse taking (77% agreement, item 1), on a campaign reporting the advantages of anticoagulation over no antithrombotic therapy (98% agreement, item 2) or of NOACs over aspirin (96% agreement, item 3) or on the practical use of NOAC (93% agreement, item 6) or on stroke and bleeding risk scores (87% agreement, item 7). See Colonna et al. (2017) [1] for further interpretation and discussion.

**Specifications Table**

**Table:** 

Subject area	*Medicine*
More specific subject area	*Use of anticoagulants in difficult cases of AF and VTE*
Type of data	*Tables, text file, list of expert, graph, figure*
How data was acquired	*Large (178 expert physicians) multidisciplinary survey; subsequent analysis according to the Delphi methodology*
Data format	*Analyzed and grouped*
Experimental factors	*Nine challenging cases of patients with atrial fibrillation and venous thromboembolism*
Experimental features	*Nine separate cases, each of them with multiple statements with 5 level of agreement to be voted by the panel of 178 expert physicians. Following analysis of concordance, according to Delphi technique.*
Data source location	*More than 150 different Italian cities where the surveys were filled in*
Data accessibility	*All the data are supplied in this article*

**Value of the data:**

• Complete data derived from 178 questionnaires filled in by Italian AF and VTE expert physicians, consisting of 9 statements of clinical complex AF and VTE cases and informative campaign on antithrombotic therapy for stroke prevention in AF.
• Data potentially valuable for the scientific community, showing the doubts of different specialists (Internists, Pneumologists, Geriatricians, Cardiologists and Neurologists) with a large experience in prescribing oral anticoagulation in difficult AF and VTE cases (see full list of participants provided).
• The data obtained in some particular clinical cases such as CHA2DS2-VASc=1, comorbid coronary artery disease, frailty, advanced age, risk of falling and prior haemorrhagic stroke, can be compared with indications from published guidelines and recommendations for future insight and to be considered as a benchmark for future trials in the area or oral anticoagulation for AF and VTE.
• The innovative data on criteria for an informative campaign on antithrombotic therapy for stroke prevention and on strategies to optimise appropriate prescription of antithrombotic therapy helps to possible new collaborations for further studies.

## 1 Data

**Fig. 1:**
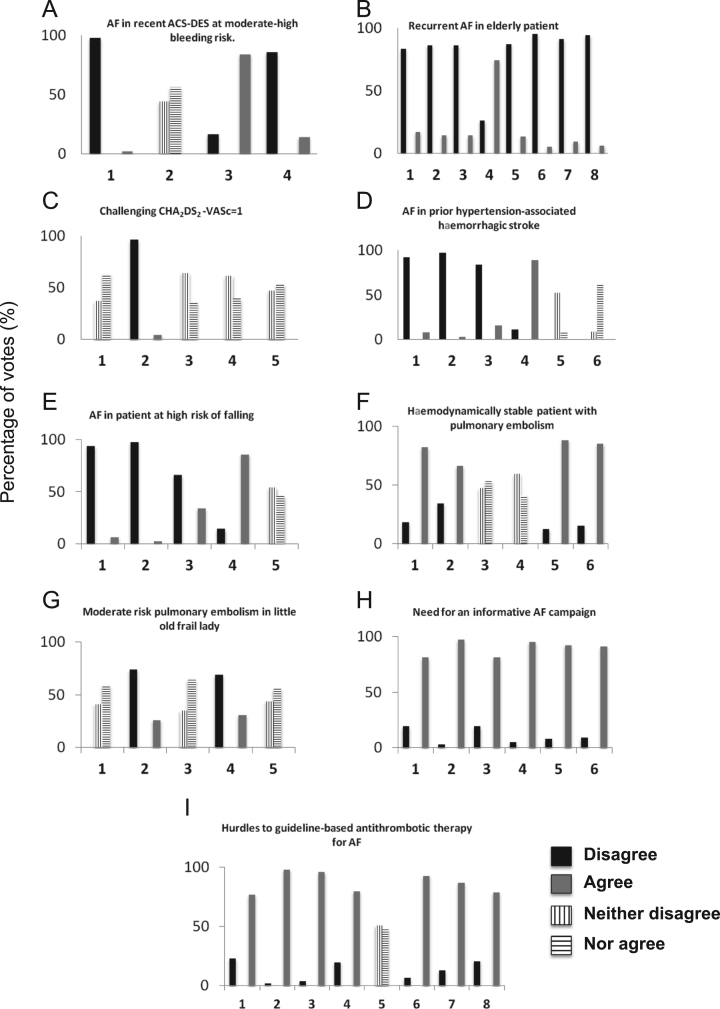
Summary of results for all statements. Distribution of the panel’s answers on the nine statements. Y-axis = percentage of panel votes; X-axis = items listed in each statement (see Ref. [1] for full explanation). ND: neither disagree; NA: nor agree.

New unreported data concerning informative campaign on antithrombotic therapy for stroke prevention (See “Statement 8” Table and Fig. 1H) showed the expert panel agreement on the inclusion of: self monitoring of heart rhythm by pulse taking in subjects older than 64 years of age (81% agreement, item 3); knowledge that the risk of stroke associated with AF is almost twice the risk associated with hypertension (95% agreement, item 4); knowledge that the CHA2DS2-VASc score exerts a higher influence on stroke risk compared to AF duration (92% agreement, item 5); knowledge that stroke prevention in AF with a NOAC is more effective, does not cause any higher bleeding risk, and is equally simple compared to aspirin treatment (91% agreement, item 6). The panel also agreed that cardioembolic stroke risk is not reduced after cardioversion from AF to sinus rhythm (81% agreement, item 1) (Ref. [2]) and that inappropriate NOAC dose reduction may reduce the efficacy in preventing thromboembolic stroke compared to the full dose regimen (97% agreement, item 2)

Data in the second Supplementary statement (see “Statement 9” Table and Fig. 1I) showed the indications of expert physicians on strategies to optimise appropriate prescription of antithrombotic therapy. The panel agreed on the utility of short television advertisements about the risks of stroke associated with AF (79% agreement, item 8), on a campaign encouraging regular control of cardiac rhythm by pulse taking (77% agreement, item 1), on a campaign reporting the advantages of anticoagulation over no antithrombotic therapy (98% agreement, item 2) or of NOACs over aspirin (96% agreement, item 3) or on the practical use of NOAC (93% agreement, item 6) or on stroke and bleeding risk scores (87% agreement, item 7). Additionally, the panel agreed on the benefits of abolishing the mandatory electronic form for NOAC prescription, restricting the use to a selected group representing a minority of guideline-eligible patients (80% agreement, item 4), while it did not reach consensus on the possibility that every physician may be allowed to prescribe a NOAC (51% ND/49% NA, item 5).

## 2 Experimental design, materials and methods

The experimental design to collect the present data is derived by the Delphi method, characterized by a large number of participants without the need for face-to-face contacts (Ref. [3]), the only choice when therapeutic decisions routine clinical practice are challenging because of lack of evidence from clinical trials in selected groups of patients, such as those with high risk of bleeding, multiple comorbidities, or receiving potentially interfering drugs.

The materials and methods consists of a Delphi Consensus panel organized to address multiple unanswered questions related to the clinical management of difficult cases of patients with AF or VTE.

We used a modified Delphi method (Refs. [4], [5]) to reach consensus in a voting panel of 178 medical doctors from different specializations (Internists, Pneumologists, Geriatricians, Cardiologists and Neurologists) with a large experience in prescribing oral anticoagulation (Ref [6]) for AF or VTE (see full list of participants included in the Appendix A of the present Data in Brief).

The following list of 9 statements on controversial topics in AF and VTE antithrombotic management was selected for the Delphi panel: 1- AF in a recent acute coronary syndrome-drug eluting stent (ACS-DES) patient at moderate-high bleeding risk; 2- Recurrent AF in an elderly patient with impaired renal function; 3- A challenging CHA_2_DS_2_ VASc (congestive heart failure, hypertension, age ≥75, diabetes, prior stroke, vascular disease, age 65–74, sex category) =1 case; 4- AF with prior hypertension-associated haemorrhagic stroke; 5- AF in a patient at high risk of falling; 6- Hemodynamically stable patient with pulmonary embolism; 7- Moderate risk pulmonary embolism in a frail lady; 8- Need for an informative AF campaign; 9- Potential hurdles to guideline-based antithrombotic therapy for AF.

Each statement was declined in 4 or more items, and each of the 178 expert physicians freely, individually, and anonymously delivered his/her level of agreement according to the following 5-point Likert scale: 1 = absolutely disagree, 2 = disagree, 3 = agree, 4 = more than agree, 5 = absolutely agree. Consensus was reached when the sum of items 1 and 2 (Disagree) or 3, 4 and 5 (Agree) reached 66%. Where no consensus was reached the data were shown as Neither Disagree/Nor Agree (ND/NA), with ND standing for the sum of items 1 and 2 and NA as the sum of items 3, 4 and 5.

Then, the experts participated in a plenary session held in Mestre, Italy, on October 5th 2016, where the data were presented and discussed. Agreement was reached on most – but not all – items. In case of no agreement, a second round of voting was purposely not performed in order to highlight the inconsistencies of opinion or the insufficient information of evidence-based literature on the therapeutic options for certain AF/VTE patients.

The Tables and the figure with the graphs from all statements are supplied to fully understand the data (Table 1, Table 2, Table 3, Table 4, Table 5). See Ref. [1] for further interpretation and discussion.

**Table 1:** AF in recent ACS-DES at moderate-high bleeding risk. Flow chart of the panel’s scores on the four listed items. Treatment with aspirin and a NOAC, according to the SmPC, is considered the most appropriate option (84%). Low NOAC dose, regardless of SmPC indications, divides the panel, with lack of consensus (44% vs 56%). The experts unanimously do not consider NOAC discontinuation (98%) or NOAC replacement with warfarin (86%) appropriate options. ACS: acute coronary syndromes; DES: drug eluting stenting; ND: neither disagree; NA: nor agree.

**Table 2:** Challenging CHA_>2_DS_2_-VASc=1. Flow chart of the panel’s scores. Four of the five therapeutic options do not reach consent. The panel unanimously disagrees with warfarin as an option (96%). ND: neither disagree; NA: nor agree.

**Table 3:** AF in prior hypertension-associated haemorrhagic stroke. Flow chart of the panel’s scores. The panel disagrees with ruling out antithrombotic therapy entirely (92%), but excludes aspirin (97%) or warfarin (84%). The panel agrees with NOAC therapy, according to the SmPC (89%), but is divided on using a low dose NOAC (52% vs 48%) or on LAA closure without any anticoagulation (39% vs 61%). LAA: left atrial appendage; ND: neither disagree; NA: nor agree.

**Table 4:** AF in an old patient at high risk of falling. Flow chart of the panel’s scores. The panel disagrees with considering the risk of falling a contraindication to antithrombotic treatment (94%). It also disagrees with aspirin (98%) or warfarin (66%) as treatment options, preferring the use of a NOAC, according to the SmPC (86%). NOAC dose reduction, regardless of SmPC indications, does not reach a consensus (54% vs 46%). ND: neither disagree; NA: nor agree.

**Table 5:** Moderate risk pulmonary embolism in a little, old, frail lady. Flow chart of the panel’s scores. The panel disagrees with prescribing a NOAC at full dose, either with parenteral treatment (74%) or after a loading dose (69%). It does not reach a consensus on prescribing parenteral therapy followed by low-dose NOAC (35% vs 65%), or parenteral therapy together with warfarin (41% vs 59%), or direct NOAC loading dose with subsequent dose reduction (44% vs 56%). ND: neither disagree; NA: nor agree. See Ref. [1] for further interpretation and discussion.
