# Supplementary material for: Data from a multidisciplinary poll of 178 expert physicians on the usage of non-vitamin K Oral Anticoagulants in patients with atrial fibrillation and venous thromboembolism
Source: Data Brief. 2017 Oct 6;15:532–9. doi: 10.1016/j.dib.2017.09.064 (PMC5651496; doi:10.1016/j.dib.2017.09.064)
Supplement: Supplementary file 2 — Supplementary material [file mmc2.pptx]

## Slide 1
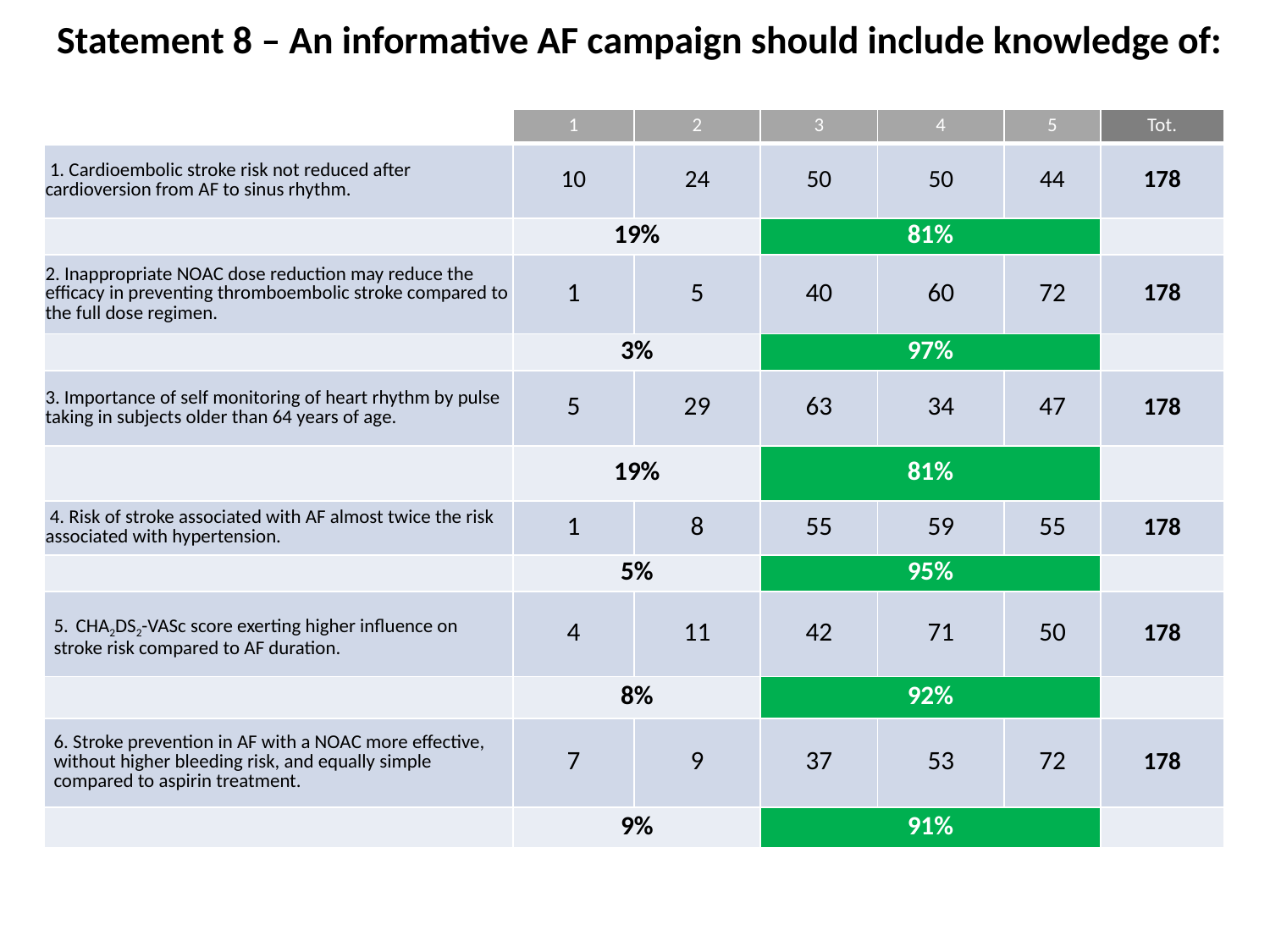

Statement 8 – An informative AF campaign should include knowledge of:
| | 1 | 2 | 3 | 4 | 5 | Tot. |
| --- | --- | --- | --- | --- | --- | --- |
| 1. Cardioembolic stroke risk not reduced after cardioversion from AF to sinus rhythm. | 10 | 24 | 50 | 50 | 44 | 178 |
| | 19% | | 81% | | | |
| 2. Inappropriate NOAC dose reduction may reduce the efficacy in preventing thromboembolic stroke compared to the full dose regimen. | 1 | 5 | 40 | 60 | 72 | 178 |
| | 3% | | 97% | | | |
| 3. Importance of self monitoring of heart rhythm by pulse taking in subjects older than 64 years of age. | 5 | 29 | 63 | 34 | 47 | 178 |
| | 19% | | 81% | | | |
| 4. Risk of stroke associated with AF almost twice the risk associated with hypertension. | 1 | 8 | 55 | 59 | 55 | 178 |
| | 5% | | 95% | | | |
| 5. CHA2DS2-VASc score exerting higher influence on stroke risk compared to AF duration. | 4 | 11 | 42 | 71 | 50 | 178 |
| | 8% | | 92% | | | |
| 6. Stroke prevention in AF with a NOAC more effective, without higher bleeding risk, and equally simple compared to aspirin treatment. | 7 | 9 | 37 | 53 | 72 | 178 |
| | 9% | | 91% | | | |
