# Supplementary material for: Data from a multidisciplinary poll of 178 expert physicians on the usage of non-vitamin K Oral Anticoagulants in patients with atrial fibrillation and venous thromboembolism
Source: Data Brief. 2017 Oct 6;15:532–9. doi: 10.1016/j.dib.2017.09.064 (PMC5651496; doi:10.1016/j.dib.2017.09.064)
Supplement: Supplementary file 3 — Supplementary material [file mmc3.pptx]

## Slide 1
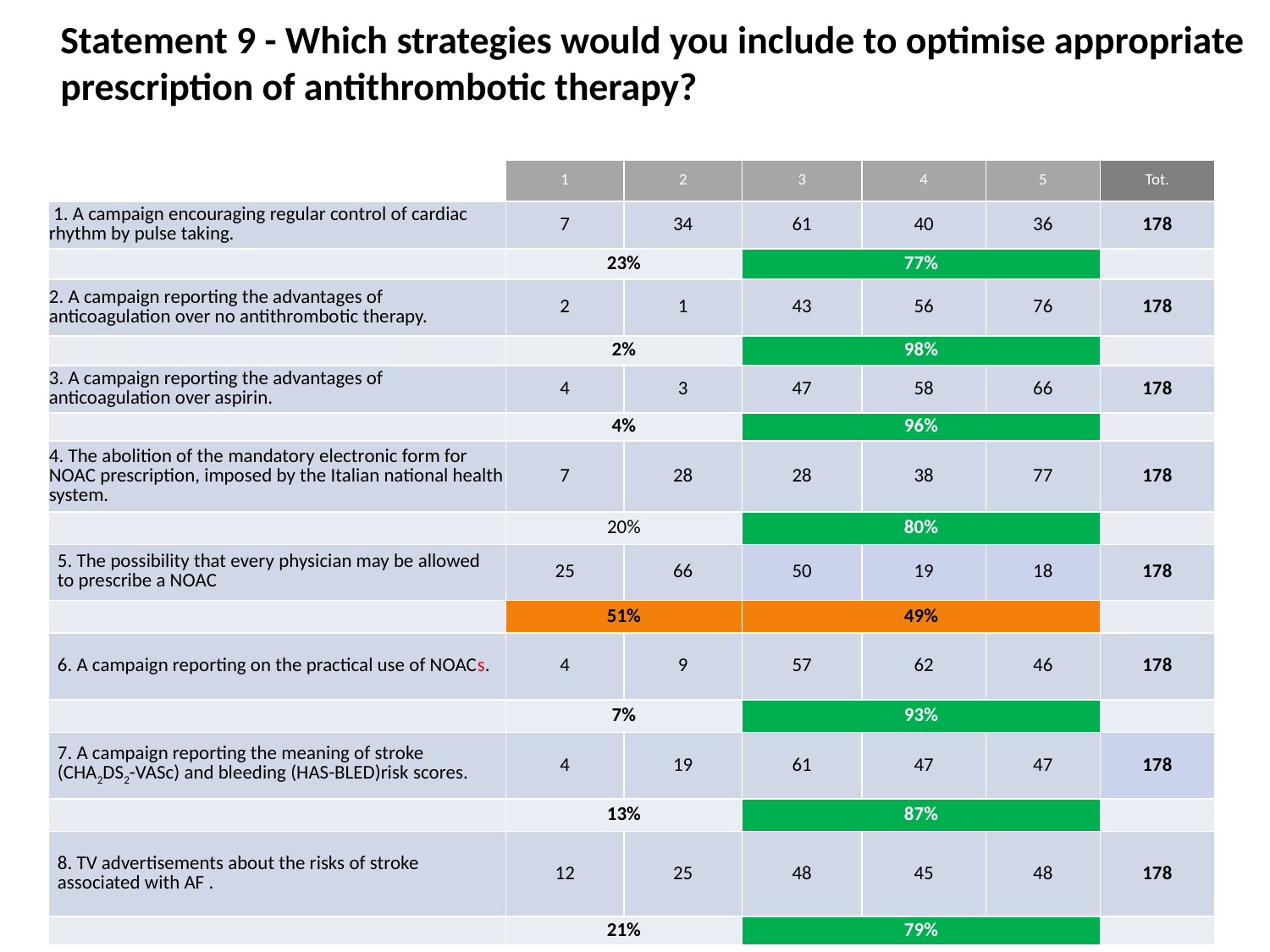

Statement 9 - Which strategies would you include to optimise appropriate prescription of antithrombotic therapy?
| | 1 | 2 | 3 | 4 | 5 | Tot. |
| --- | --- | --- | --- | --- | --- | --- |
| 1. A campaign encouraging regular control of cardiac rhythm by pulse taking. | 7 | 34 | 61 | 40 | 36 | 178 |
| | 23% | | 77% | | | |
| 2. A campaign reporting the advantages of anticoagulation over no antithrombotic therapy. | 2 | 1 | 43 | 56 | 76 | 178 |
| | 2% | | 98% | | | |
| 3. A campaign reporting the advantages of anticoagulation over aspirin. | 4 | 3 | 47 | 58 | 66 | 178 |
| | 4% | | 96% | | | |
| 4. The abolition of the mandatory electronic form for NOAC prescription, imposed by the Italian national health system. | 7 | 28 | 28 | 38 | 77 | 178 |
| | 20% | | 80% | | | |
| 5. The possibility that every physician may be allowed to prescribe a NOAC | 25 | 66 | 50 | 19 | 18 | 178 |
| | 51% | | 49% | | | |
| 6. A campaign reporting on the practical use of NOACs. | 4 | 9 | 57 | 62 | 46 | 178 |
| | 7% | | 93% | | | |
| 7. A campaign reporting the meaning of stroke (CHA2DS2-VASc) and bleeding (HAS-BLED)risk scores. | 4 | 19 | 61 | 47 | 47 | 178 |
| | 13% | | 87% | | | |
| 8. TV advertisements about the risks of stroke associated with AF . | 12 | 25 | 48 | 45 | 48 | 178 |
| | 21% | | 79% | | | |
